# Type 2 Diabetes in a Portuguese Adolescent With Hijazi-Reis Syndrome

**DOI:** 10.7759/cureus.100610

**Published:** 2026-01-02

**Authors:** Maria Inês Neto, Francisco Baptista, Marta Zegre Amorim, Isabel Bretes, Susana Correia

**Affiliations:** 1 Pediatrics Department, Hospital Nossa Senhora do Rosário, Unidade Local de Saúde do Arco Ribeirinho, Barreiro, PRT; 2 Medical Genetics Department, Hospital Lusíadas Lisboa, Lisboa, PRT

**Keywords:** case report, diabetes mellitus type 2, neurodevelopmental disorders, pediatric obesity, tceal1 protein, x-linked intellectual disability

## Abstract

Hijazi-Reis syndrome is a recently described, rare, X-linked neurodevelopmental disorder caused by loss-of-function variants in the *TCEAL1* gene. Fewer than 15 cases have been reported to date, with key features including developmental delay, especially affecting expressive speech, intellectual disability, autistic traits, and variable systemic findings.

We describe the case of a Portuguese girl with severe intellectual disability, absent speech, autistic traits, obesity, and mild dysmorphic facial features. Genetic testing identified a *de novo* exon 3 deletion in *TCEAL1*, confirming the diagnosis of Hijazi-Reis syndrome. At age 14, she developed type 2 diabetes with preserved insulin secretion and negative autoimmune markers.

This first Portuguese case adds to the limited clinical descriptions of Hijazi-Reis syndrome and documents the occurrence of type 2 diabetes in an affected adolescent. As further cases are reported, consideration of metabolic aspects may support comprehensive follow-up and multidisciplinary care in affected individuals.

## Introduction

Hijazi-Reis syndrome is a recently described neurodevelopmental disorder caused by loss-of-function variants in the *TCEAL1* gene (Xq22.2). Fewer than 15 cases of this monogenic disorder have been reported to date [1-3].

The *TCEAL1* (transcription elongation factor A-like 1) gene encodes a nuclear phosphoprotein involved in transcriptional regulation, critical for normal neuronal development and gene expression regulation. Loss-of-function variants in this gene lead to impaired neuronal differentiation and synaptic function, which underlie the neurodevelopmental phenotype observed in affected individuals [1,4-6].

The syndrome is primarily characterized by global developmental delay and severe intellectual disability with a particular impact on expressive language, autistic-like behaviors, hypotonia, abnormal gait, and mildly dysmorphic facial features. Additional reported features include ocular anomalies (strabismus, refractive errors, nystagmus), gastrointestinal abnormalities (gastroesophageal reflux, constipation, dysmotility), recurrent infections, and seizures [1,7]. Brain imaging is abnormal in about 50% of patients, with common findings including myelination defects and structural brain anomalies such as subcortical heterotopia and corpus callosum abnormalities [1,3].

We present the case of a Portuguese girl with a *de novo* pathogenic *TCEAL1* variant, adding further clinical detail to the limited literature on Hijazi-Reis syndrome. Parental consent was obtained for the publication of this case report.

## Case presentation

We report the case of a 14-year-old girl under multidisciplinary follow-up at the pediatric department of a Portuguese district hospital, primarily due to neurodevelopmental issues.

She was born at term to healthy, non-consanguineous parents after an uneventful pregnancy. The Apgar score was 9/10, and the birth weight was 2515 g (appropriate for gestational age). Early development was reportedly normal until 10-12 months, when her parents started noticing some developmental delays. She began walking at 17 months, spoke her first words at 15 months with minimal further progression, and did not engage in playful social interactions.

Over time, the developmental delay progressed to a severe intellectual disability, with no functional language (limited to 10-20 isolated words or syllables without context), requiring intensive and permanent support and care across all domains of adaptive functioning. From early childhood, the patient was followed by psychology and child psychiatry services and received multidisciplinary support, including early intervention programs and occupational therapy, to address developmental and behavioral difficulties.

Behavioral disturbances included irritability, mood swings, inattention, periods of agitation with self- and hetero-aggressive behavior, sleep difficulties, daytime bruxism, and compulsive eating behaviors characterized by impaired satiety and recurrent food-seeking. At six years of age, a formal cognitive assessment, along with the ADI-R and ADOS-2, revealed severe cognitive impairment and marked deficits in social and adaptive functioning.

**Figure 1:**
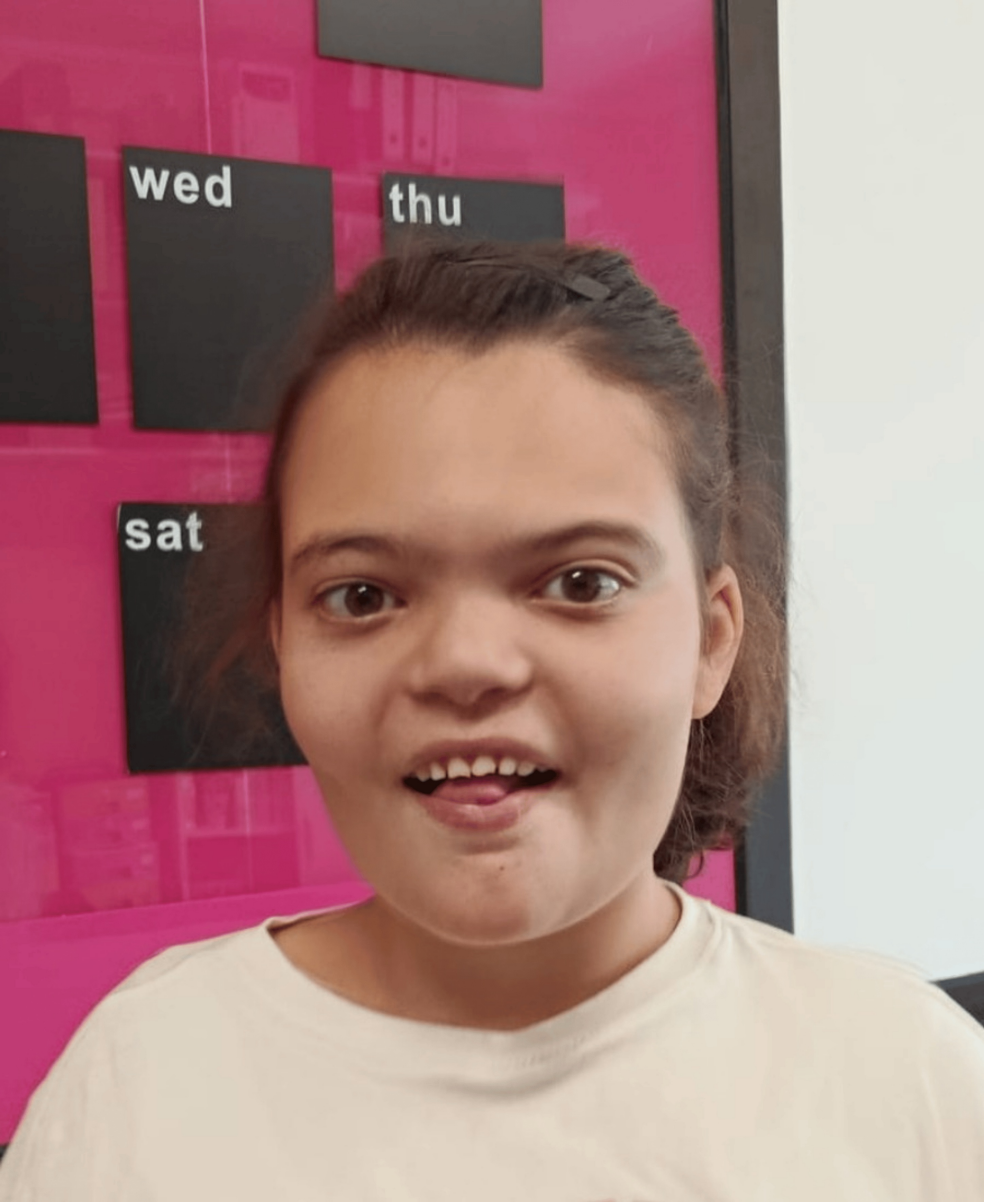
Characteristic facial features of the patient at age 14, including mild synophrys, depressed nasal bridge, smooth philtrum, tented upper lip, notched, widely spaced teeth, and mild right eye esotropia Parental informed consent was obtained for the publication of this image.

Additional features included dysmorphic traits, such as mild synophrys, depressed nasal bridge, smooth philtrum, tented upper lip, and notched, widely spaced teeth (Figure 1), as well as height at the lower limit of normal (5th percentile at 14 years, WHO growth charts) and obesity (BMI 26.2 kg/m², 95th percentile). The neurological examination showed generalized joint hyperlaxity, without motor asymmetries or ataxia. Deep tendon reflexes were present and symmetrical.

The brain MRI revealed a small signal defect in the left centrum semiovale, with characteristics similar to the cerebral cortex and lenticular nucleus, suggestive of a neuronal migration defect versus hamartoma. The patient was evaluated by an inherited metabolic diseases specialist to exclude inborn errors of metabolism.

Given the syndromic presentation, she was referred for genetic evaluation. Karyotype and chromosomal microarray were normal for a female. A multigene panel for intellectual disability and subsequent Mendeliome reanalysis identified only variants of uncertain significance (VUS), with segregation analysis not altering their classification. Trio whole-exome sequencing revealed a heterozygous *de novo* deletion in the X chromosome, described as NC_000023.10:g.(?102884815)(102885354_?)del, involving exon 3 of the *TCEAL1* gene, classified as likely pathogenic and consistent with the clinical phenotype, thus establishing the molecular diagnosis of Hijazi-Reis syndrome.

At age 14, she was diagnosed with diabetes (HbA1c 8.4%), hypercholesterolemia, and hypertriglyceridemia (total cholesterol 208 mg/dL, triglycerides 186 mg/dL). Autoimmunity testing for type 1 diabetes was negative, including anti-insulin, anti-pancreatic islet, anti-IA-2 (tyrosine phosphatase), anti-ZnT8 (zinc transporter 8), and anti-GAD2 (glutamic acid decarboxylase) antibodies. Genetic testing for maturity-onset diabetes of the young (MODY) was also negative. The C-peptide level was 3.3 ng/mL, indicating preserved endogenous insulin secretion. Laboratory values at diagnosis are detailed in Table 1. These findings were considered most consistent with type 2 diabetes, and she was started on metformin, titrated to 850 mg twice daily, and referred to a nutritionist for dietary optimization.

**Table 1:** Metabolic and autoimmune laboratory findings at diagnosis HbA1c: glycated hemoglobin, LDL: low-density lipoprotein cholesterol, HDL: high-density lipoprotein cholesterol

Test (Units)	Result	Reference value
HbA1c (%)	8.4	<5.7
Total cholesterol (mg/dL)	208	<170
LDL-Cholesterol (mg/dL)	148	<110
HDL-Cholesterol (mg/dL)	23	>45
Triglycerides (mg/dL)	186	<90
Anti-insulin antibodies (UA/mL)	<8.0	Negative: <20
Islet cell antibodies, IgG (U/mL)	4.2	Negative: <28
Anti–tyrosine-phosphatase protein antibodies (IA-2) (U/mL)	<0.8	Negative: <1
Zinc transporter 8 antibodies (ZnT8) (U/mL)	<10	Negative: <10
GAD 2 antibodies (anti–glutamic acid decarboxylase 65 kD) (U/mL)	<0.7	Negative: <1
C-peptide (ng/mL)	3.3	0.78–5.19

## Discussion

We report the first Portuguese case of Hijazi-Reis syndrome due to a *de novo* deletion encompassing exon 3 of the *TCEAL1* gene.

Previous studies by Hijazi et al. and Yamamoto et al. helped define the spectrum of neurodevelopmental phenotypes associated with Xq22 deletions, which may encompass *TCEAL1* and adjacent genes. These deletions were initially described in females, with neurological features ranging from severe intellectual disability and hypotonia to behavioral abnormalities. These studies narrowed the smallest region of overlap to a six-gene interval, implicating *TCEAL1* as a key contributor to the phenotype. At the molecular level, *TCEAL1* haploinsufficiency likely disrupts transcriptional regulation and neuronal development. While deletion size does not strictly predict clinical severity, variability appears to depend on the specific gene content and X-inactivation patterns in affected individuals [1,3,5,6].

This case adds to the currently limited body of literature and underscores the importance of comprehensive genetic testing in children with unexplained syndromic intellectual disability. Whole-exome or genome sequencing can identify rare monogenic disorders and genetic syndromes, clarifying overlapping features, such as intellectual disability, facial dysmorphism, brain malformations, and behavioral disturbances, in line with current expert and guideline recommendations [8,9].

Our patient exhibited several features consistent with previous descriptions, including global developmental delay, joint hyperlaxity, and delayed ambulation, evolving to severe intellectual disability with marked expressive language deficits, impaired adaptive and social functioning, and behavioral abnormalities such as emotional outbursts [1,3,7]. These findings align with the phenotypic spectrum delineated by Hijazi et al. (2022) and subsequently refined by Albuainain et al. (2024) [1,3].

Although ataxia and seizures have been reported in some affected individuals, these manifestations were absent in our patient. Recurrent infections, occasionally noted in prior cohorts, were also not observed. Although refractive errors, strabismus, nystagmus, and other ocular anomalies have been reported in Hijazi-Reis syndrome, no formal ophthalmologic assessment was performed in our patient [1,3]. 

The brain MRI revealed a small gray-matter-like lesion in the left centrum semiovale, suggestive of a subcortical heterotopia. This finding is consistent with the neuronal migration abnormalities previously reported in Hijazi-Reis syndrome, in which abnormal myelination, subcortical heterotopia, and corpus callosum anomalies were the main neuroimaging findings [1,3].

Craniofacial dysmorphism in our patient included mild synophrys, a depressed nasal bridge, smooth philtrum, and a tented upper lip, features overlapping with those described by Hijazi et al. (2022), Albuainain et al. (2024), and Yamamoto et al. (2024), who reported facial coarsening, thin or bow-shaped upper lips, broad nasal bridge and long philtrum in affected individuals. These subtle but consistent findings support the presence of a recognizable, though variable, facial gestalt in this disorder [1-3].

To the best of our knowledge, this is the first reported case of type 2 diabetes in a Hijazi-Reis syndrome patient. Endocrinological abnormalities have been variably reported in affected individuals, including growth retardation, obesity (affecting approximately 50% of patients), premature or delayed puberty, hyperandrogenemia, polycystic ovary syndrome (PCOS), hyperinsulinemia, and, in at least one case, hypertriglyceridemia [1,3].

Interestingly, Albuainain et al. (2024) noted that these traits appear to affect females more frequently than males, although small cohort sizes limit firm sex-specific conclusions. Some patients with short stature or growth retardation have shown low insulin-like growth factor (IGF)-1 levels, further supporting a possible endocrine involvement in the disorder [3].

The endocrine profile observed in Hijazi-Reis syndrome is consistent with patterns seen in pediatric insulin-resistance and monogenic obesity disorders. Obesity-driven insulin resistance is a key contributor to hyperandrogenemia and PCOS in adolescent girls and can worsen ovarian dysfunction. Hyperinsulinemia, frequently reported in syndromic neurodevelopmental disorders, represents an early marker of metabolic dysregulation and a known precursor to type 2 diabetes [10,11].

Although type 2 diabetes has not previously been reported in Hijazi-Reis syndrome, its occurrence in our patient can be interpreted in the context of clinical features that overlap with those observed in other syndromic obesity conditions. Hyperphagia, compulsive eating behaviors, and obesity are known contributors to insulin resistance and increased diabetes risk. In this setting, diabetes in our patient may represent a metabolic consequence of these factors rather than an unrelated comorbidity, although no causal association can be established based on a single observation, and further clinical data are required [12,13].

## Conclusions

This case adds an additional clinical observation to the limited literature on Hijazi-Reis syndrome and highlights the presence of metabolic and endocrinological findings in an affected individual. The occurrence of type 2 diabetes in the context of compulsive eating behavior and obesity, characteristics seen in other syndromic conditions, underscores the importance of careful metabolic surveillance.

Continued reporting, long-term clinical follow-up, and further clinical characterization of affected individuals will be essential to refine genotype-phenotype correlations and clarify the natural history of Hijazi-Reis syndrome. Awareness of potential metabolic and endocrine aspects supports the need for comprehensive, multidisciplinary care to optimize outcomes.

## References

[1] Hijazi H, Reis LM, Pehlivan D (2022). TCEAL1 loss-of-function results in an X-linked dominant neurodevelopmental syndrome and drives the neurological disease trait in Xq22.2 deletions. Am J Hum Genet.

[2] Shimojima Yamamoto K, Itagaki Y, Tanaka K, Okamoto N, Yamamoto T (2024). Xq22 deletion involving TCEAL1 in a female patient with early-onset neurological disease trait. Hum Genome Var.

[3] Albuainain F, Shi Y, Lor-Zade S (2024). Confirmation and expansion of the phenotype of the TCEAL1-related neurodevelopmental disorder. Eur J Hum Genet.

[4] Pillutla RC, Shimamoto A, Furuichi Y, Shatkin AJ (1999). Genomic structure and chromosomal localization of TCEAL1, a human gene encoding the nuclear phosphoprotein p21/SIIR. Genomics.

[5] Yamamoto T, Wilsdon A, Joss S (2014). An emerging phenotype of Xq22 microdeletions in females with severe intellectual disability, hypotonia and behavioral abnormalities. J Hum Genet.

[6] Hijazi H, Coelho FS, Gonzaga-Jauregui C (2020). Xq22 deletions and correlation with distinct neurological disease traits in females: further evidence for a contiguous gene syndrome. Hum Mutat.

[7] Kniffin CL. HIJAZI-REIS SYNDROME; HIJRS. Online Mendelian Inheritance in Man (OMIM (2025). Hijazi-Reis syndrome; HIJRS. https://www.omim.org/entry/301094?search=hijazi-reis&highlight=%22hijazi%20rei%22%2Chijazirei.

[8] Tolezano GC, Bastos GC, da Costa SS (2024). Clinical characterization and underlying genetic findings in Brazilian patients with syndromic microcephaly associated with neurodevelopmental disorders. Mol Neurobiol.

[9] Rodan LH, Stoler J, Chen E, Geleske T (2025). Genetic evaluation of the child with intellectual disability or global developmental delay: clinical report. Pediatrics.

[10] Barber TM, Franks S (2021). Obesity and polycystic ovary syndrome. Clin Endocrinol (Oxf).

[11] Prosperi S, Chiarelli F (2025). Insulin resistance, metabolic syndrome and polycystic ovaries: an intriguing conundrum. Front Endocrinol (Lausanne).

[12] Janssen JA (2022). New insights into the role of insulin and hypothalamic-pituitary-adrenal (HPA) axis in the metabolic syndrome. Int J Mol Sci.

[13] Silva-Júnior AE, Macena ML, Bueno NB (2025). The prevalence of food addiction and its association with type 2 diabetes: a systematic review with meta-analysis. Br J Nutr.

